# Active practice charter accreditation is associated with higher quality outcomes in English general practice: a cross-sectional observational study

**DOI:** 10.1186/s12875-026-03272-y

**Published:** 2026-03-29

**Authors:** Jerome Mayaud, Callum Leese, Rosina Cross, Robert Mann, Emma Cockroft

**Affiliations:** 1 Health & Community Sciences Department, University of Exeter, Exeter, UK; 2 Population Health and Genomics Department, University of Dundee, Dundee, UK; 3 Medical School Primary Care Department, University of Exeter, Exeter, UK; 4 Department of Public Health and Sport Sciences, University of Exeter, Exeter, UK

**Keywords:** Primary care, Social prescribing, Health equity, Socioeconomic deprivation, Quality improvement

## Abstract

**Background:**

The Active Practice Charter (APC), introduced by the Royal College of General Practitioners (RCGP), aims to embed physical activity (PA) promotion within primary care, theoretically enhancing both patient health and practice quality. However, the effectiveness of this voluntary accreditation and its social equity implications remain under-researched. This study aims to assess the independent relationship between APC accreditation and three quality outcomes (Quality and Outcomes Framework (QOF) score, patient satisfaction rate and Care Quality Commission (CQC) rating) while exploring whether this relationship varied across the socioeconomic deprivation spectrum. This is the first large-scale empirical assessment of the APC.

**Methods:**

This cross-sectional observational study analysed data from 6,063 General Practice (GP) practices in England (523 APC-accredited). Initial non-parametric bivariate tests showed APC practices tended to have larger list sizes, were located in less socioeconomically deprived areas, and served a greater proportion of older adults than non-APC practices. Multivariable regression (Ordinary Least Squares and Ordinal Logistic) was used to assess the main effect of APC status, controlling for size, socioeconomic deprivation, and patient demographics. Equity implications were explored through moderation analysis and stratification analysis across ten deciles of the Index of Multiple Deprivation (IMD).

**Results:**

When compared with non-APC practices, APC practices had statistically significantly lower IMD scores, higher proportions of older adults and a higher proportion of White ethnicity. This implies some socioeconomic inequities in the take-up of the APC programme across England. In our main effects model, APC accreditation had a statistically significant and robust positive association with patient satisfaction (β = 1.979, *p* < 0.001) and CQC ratings (OR = 1.321, *p* < 0.001), and only a small but significant association with QOF points (β = 0.893, *p* < 0.01). Moderation analysis suggested uniform effects across the socioeconomic deprivation spectrum, although followup stratification analysis revealed some evidence of social inequity, with the positive effects on patient satisfaction being concentrated in the mid-range of socioeconomic deprivation.

**Conclusions:**

APC accreditation is associated with higher levels of patient experience and regulatory success, strengthening the case for its promotion. There was evidence of socioeconomic patterning in APC uptake, but no statistically significant global interaction in outcomes by deprivation. Future policy should focus on providing dedicated resources and tailored support to overcome resource constraints in socioeconomically deprived settings to ensure the APC does not inadvertently widen existing quality gaps.

## Background

In 2018, the Royal College of General Practitioners (RCGP) introduced the Active Practice Charter (APC) as an initiative to champion physical activity (PA) and reduce sedentary behaviour within primary care settings across the United Kingdom [1]. PA can have significant physical, psychological, and social health benefits [2, 3], which is why it is recognised by the World Health Organisation as a key factor in both the prevention and management of long-term health conditions [4]. The burden of physical inactivity is significant in the UK: it is associated with 1 in 6 deaths, and is estimated to cost the country £7.4 billion annually, including £0.9 billion to the National Health Service (NHS) [5].

There are large disparities in PA participation rates in relation to age, socioeconomic deprivation, geography, comorbidity, ethnic group, and gender [6–9]. Primary care has been identified as a useful tool in reaching groups with lower PA, most notably those with comorbidities [10], with evidence supporting the effectiveness of PA promotion in primary care [11, 12]. Despite this, PA promotion in primary care settings is infrequently delivered owing to a large number of barriers, including time, resources and knowledge [13, 14].

The RCGP developed the APC to support primary care professionals to overcome these barriers. General practice (GP) practices aspiring to APC accreditation commit to implementing active changes across the following five key criteria [1, 15]: (1) reducing sedentary behaviour in staff; (2) reducing sedentary behaviour in patients; (3) increasing PA in staff; (4) increasing PA in patients; and (5) fostering partnerships with local PA providers.

By seeking to enhance wellbeing, cohesion and morale among staff, the APC could further improve patient experiences and practice functioning. The emphasis on team-based activity and community partnership may also strengthen local engagement, which could impact patient satisfaction and service delivery. These mechanisms provide a plausible pathway through which APC accreditation could be associated with broader indicators of quality [16].

However, there is no funding available to support the delivery of the APC at a practice level, with the scheme being implemented on a voluntary basis. It is recognised that voluntary schemes are less likely to be implemented in practices in areas of high socioeconomic deprivation, given the increased demands and pressure on these services [17–19]. This may be compounded by the APC, as lifestyle-based initiatives also carry an inherent risk of unintentionally exacerbating existing health inequalities [20–22].

### Research aim and objectives

Based on the gaps in the literature, the aim of this study is to assess the independent relationship between APC accreditation and three quality outcomes: Quality and Outcomes Framework (QOF) score, patient satisfaction rate, and Care Quality Commission (CQC) rating.

To achieve this research aim, we outline three objectives. Objective one is to explore the differences in sociodemographic characteristics (age, sex, ethnicity, English Indices of Multiple Deprivation (IMD) score, practice list size and practice geography) between APC practices and non-APC practices. Objective two is to model the relationship between APC accreditation and the three stated quality outcomes. Objective three is to assess whether the effect of APC status on quality outcomes differs significantly across the spectrum of deprivation.

## Methods

### Study design and setting

This study used a cross-sectional, observational design drawing on routinely collected, publicly available datasets to examine associations between APC accreditation and indicators of GP quality in England. Data were included only from GP practices located in England (i.e. excluding Wales, Northern Ireland and Scotland) owing to data completeness, since linkable datasets with relevant outcome measures are collected by Public Health England.

**Fig. 1:**
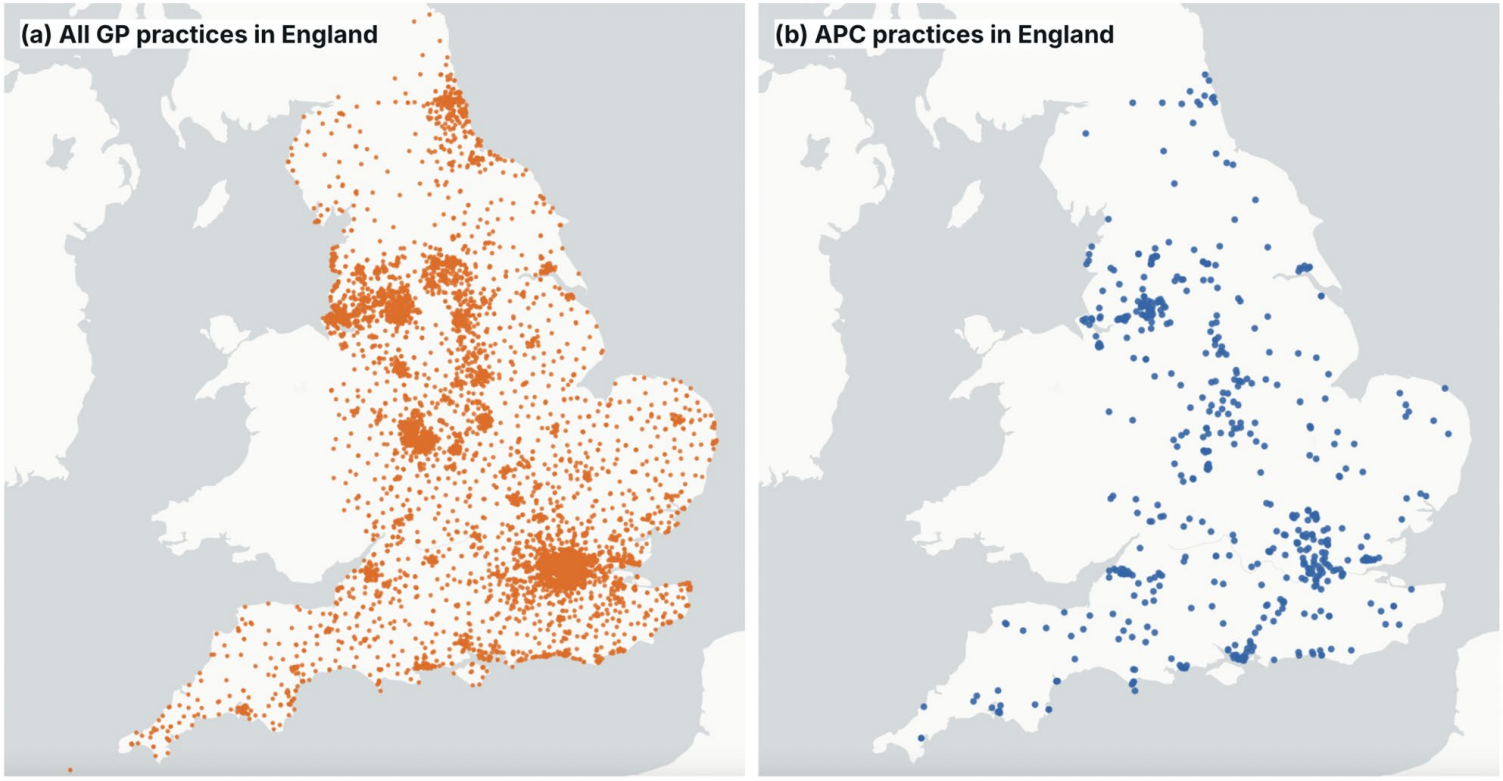
Locations of GP practices in England included in this study: **a** all practices in orange; **b** APC practices only in blue

As of September 2025, there were 6,229 GP practices in England [23], of which 551 were APC practices [24]. Our final sample, once data were cleaned and practices with incomplete data were removed, contained 6,063 practices, of which 523 (8.6%) were APC practices and 5,540 (91.4%) were non-APC practices. Figure 1 shows the locations of all the practices analysed in this study.

### Datasets and variables

The independent and outcome variables used in this study are listed in Table 1. All datasets were ultimately linked to individual GP practices via their unique General Medical Practice Codes. We used the most recent publicly available datasets available for each variable; while there were slight discrepancies between the time periods at which different variables were measured, we used the same time period consistently within each variable (except for the CQC ratings dataset). While the Indices of Multiple Deprivation (IMD) dataset predates most of the other datasets in our study, it remains one of the most well-established and commonly used measures of socioeconomic deprivation in England, and we consider it appropriate for our study. For CQC ratings, there was a wide distribution of dates for which the most recent rating was available for each practice, ranging from 2015 to 2024 (Appendix 1). We conducted sensitivity analysis (reported in the Results) to assess this potential discrepancy between CQC rating and APC accreditation.

**Table 1:** Key characteristics of variables used in this study

Variable type	Variable	Data source	Data time period	Data type	Description
Independent	Practice APC status	RCGP Active Practice Charter List	Sep 2025	Binary (0 = No, 1 = Yes)	Whether or not a GP practice has APC accreditation.
Independent	IMD composite score	English Index of Multiple Deprivation (IMD)	2019	Numerical	An overall measure of socioeconomic deprivation at the LSOA level, combining 7 measures of different deprivation domains.
Independent	IMD decile	English Index of Multiple Deprivation (IMD)	2019	Ordinal (0–10)	Within-sample calculation of IMD decile based on raw IMD data.
Independent	Practice list size	Quality and Outcomes Framework (QOF)	2022–2023	Numerical	Number of people registered to the GP surgery. Accounts for structural differences between practices.
Independent	Practice geography	Rural Urban Classification (RUC)	2025	Categorical (Urban; Rural)	Standardised method for categorising LSOA geographies as either rural or urban, based on address density, physical settlement form, population size and relative access to major towns and cities.
Independent	Age group count	Practice Age and Gender Distribution	2024	Categorical (Children = 0–9 yrs; Adolescents = 10–19 yrs; Adults = 20–64 yrs; Older adults = 65 + yrs)	Count of patients at each practice, by age group (genders and ethnicity combined).
Independent	Sex count	Practice Age and Gender Distribution	2024	Categorical (Female; Male)	Count of patients at each practice, by sex (ages and ethnicity combined).
Independent	Ethnicity count	Practice Age and Gender Distribution	2024	Categorical (White; Asian / Asian British; Black; Mixed; Other).	Count of patients at each practice, by ethnicity (ages and gender combined).
Outcome	Patient satisfaction score	GP Patient Survey (GPPS)	2023	Numerical (%)	% who have a positive experience of their GP practice. Direct measure of patient experience and overall perception of care quality.
Outcome	Total QOF points	Quality and Outcomes Framework (QOF)	2022–2023	Numerical	Comprehensive indicator of clinical performance across various disease areas and public health.
Outcome	CQC rating	Care Quality Commission (CQC) Ratings	2015–2024	Ordinal (1 = Inadequate; 2 = Requires improvement; 3 = Good; 4 = Outstanding)	Independent regulatory assessment of safety, effectiveness, caring, responsiveness, and leadership.

An up-to-date list of all APC-accredited practices in England was provided by the RCGP, as of late September 2025.

While the original IMD scores are calculated at the Lower Layer Super Output Area (LSOA) level, the Office for Health Improvement and Disparities (OHID) adapts them for use at the GP level by taking a population-weighted average of the IMD scores for all LSOAs where a practice’s registered patients live. We used OHID’s adapted scores to ensure an accurate reflection of socioeconomic deprivation experienced by the population served by each practice. To provide flexibility in our analysis, and to allow us to handle potential non-linear relationships, we also calculated IMD deciles from our final study sample. This involved splitting the IMD scores into ten distinct categories, from ‘most deprived’ (decile 1) to ‘least deprived’ (decile 10).

Maintaining some measure of age and ethnicity distribution helps us account for demographic differences that might influence patient outcomes, as well as the relevance and uptake of APC initiatives by each practice’s patient population. We amalgamated 5-year age group categories reported by the OHID into four age brackets, as recommended by [25], to generate meaningful statistical analyses: (1) children (0–9 yrs); (2) adolescents (10–19 yrs); (3) adults (20–64 yrs); and (4) older adults (65 + yrs). The raw distribution of the original 5-year age group categories are presented in Appendix 2.

We combined the ethnicity classifications (*n* = 20) in the UK census into five categories, as recommended by the Office for National Statistics [26], to simplify our analysis: (1) White; (2) Asian / Asian British; (3) Black; (4) Mixed; and (5) Other.

QOF participation is practice-led and partly voluntary, although participation rates are consistently high (e.g. 97.5% of practices in England participated in 2022–2023).

For the patient experience outcome, we used the 2023 GP Practice Survey dataset (the latest available from OHID) to extract the proportion of respondents who reported a positive experience of their GP practice. This patient satisfaction score is a direct measure of patient experience and overall perception of care quality.

The CQC is the independent regulator of health and adult social care in England, and its scoring framework represents an external validation process in quality outcomes. CQC ratings use a four-point scale, from ‘Outstanding’ to ‘Inadequate’. We excluded any practices (*n* = 231) from our study sample whose rating was not available – missing ratings can occur for several reasons, including that some services are exempt from rating, the service has not yet been inspected, or the rating was suspended.

### Statistical analysis

#### Descriptive statistics

We summarised the characteristics of APC practices versus non-APC practices across all independent and outcome variables. This involved calculating averages, standard deviations, and ranges as appropriate for continuous variables, and frequencies and percentages for categorical variables. The distribution of all continuous variables were assessed for normality, skewness, and kurtosis to inform the appropriate choice of subsequent statistical modelling.

#### Bivariate analyses

Crude associations between APC status and each quality outcome measure were initially explored through bivariate analyses: Mann-Whitney U tests for continuous variables, and Chi-squared tests for categorical variables. These tests provided initial insights into potential relationships without controlling for confounders.

#### Multivariable regression modelling

Multivariable regression models were used to assess the independent association between APC status and each quality outcome, systematically controlling for identified confounding variables. We used Ordinary Least Squares (OLS) linear regression models for continuous outcome variables (QOF points and patient satisfaction scores), and an ordinal logistic regression (OLR) model for the ordinal outcome variable (CQC rating). A Brant test was performed to verify the proportional odds assumption for OLR. The test indicated that the strength of the association varied only slightly across thresholds (β = 0.78–0.87) and was consistently positive; we therefore consider the OLR as a parsimonious summary of the relationship. The results of the ordinal logit model are presented as odds ratios (OR), which have a consistent interpretation across all possible boundaries in the CQC scale (i.e. they represent the change in the odds of a practice achieving a superior CQC rating, for a one-unit increase in the predictor variable).

In order to avoid potential issues owing to highly correlated variables in our models, we performed iterative variance inflation factor (VIF) analysis to progressively reduce the number of variables in our models. This ensured an optimal balance between model power and multicollinearity. We examined pairwise correlations and calculated VIF, considering any variable pair with a coefficient greater than 0.6 to be highly correlated. For each highly correlated pair, we retained the variable that offered the better goodness-of-fit statistics and excluded its counterpart from the final model. We continued until all remaining variables had an acceptable (< 5) VIF. Following this iterative VIF analysis, we reduced our model parameter set to APC status, IMD score, list size, urban-rural, proportion of female patients, proportion of older adults and White ethnicity proportion. We excluded other age group and ethnicity categories.

#### Social equity analysis

To determine whether the effect of APC status on quality outcomes significantly differs across the spectrum of socioeconomic deprivation, we conducted moderation analysis by introducing a new variable to act as a moderator in our regression models: IMD score x APC status. Statistically significant interaction would indicate that the relationship between APC status and the outcome is not constant, and therefore inequitably distributed. To further explore potential inequities, we stratified our social equity analysis by testing the independent effect of APC status on each outcome variable within each IMD decile.

### Hypotheses

We present three hypotheses to test, relating to each of the study objectives:

- Objective 1: We hypothesise that APC practices are more commonly located in less socioeconomically deprived areas, where the GP-to-patient ratio is typically higher.
- Objective 2: We hypothesise that APC practices have better quality outcomes than non-APC practices, controlling for the sociodemographic variables outlined in Objective 1.
- Objective 3: We hypothesise that gains in quality outcomes associated with APC accreditation are inequitably distributed, such that the gains are non-uniform across the IMD deciles.

## Results

### Characteristics of APC and non-APC practices

#### Socioeconomic characteristics

**Fig. 2:**
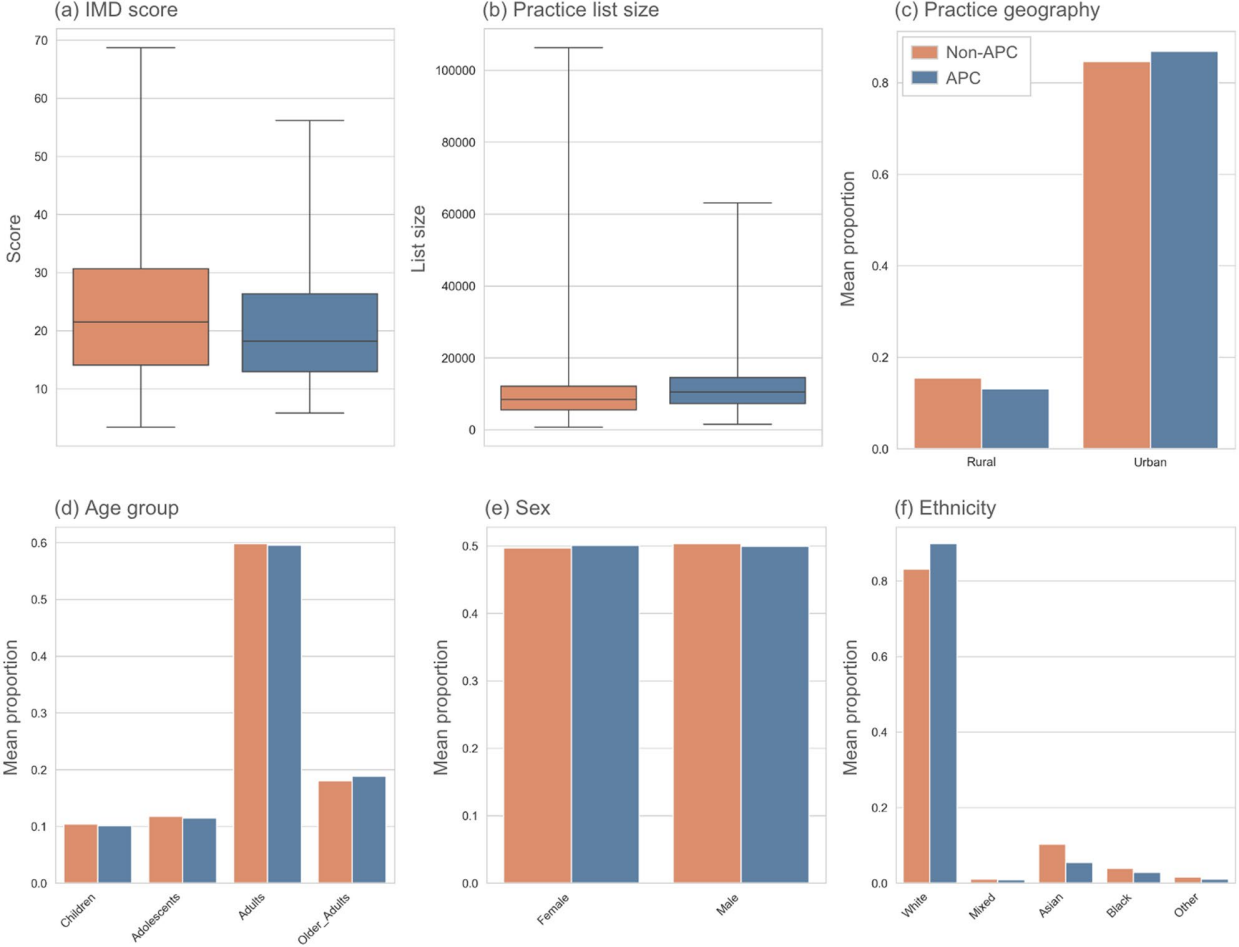
Distribution of independent variables, comparing APC (blue) and non-APC (orange) practices: **a** IMD score; **b** practice list size; **c** practice geography; **d** age group; **e** sex; and **f** ethnicity. Box-and-whisker plots in panels (**a**) and (**b**) display median, upper and lower quartile as box, and minimum and maximum values as whiskers

The distribution of the six independent variables chosen for this study are shown in Fig. 2. Result summaries from non-parametric Mann-Whitney U tests (to detect differences between APC and non-APC practices) are presented in Table 2.

**Table 2:** Mann-Whitney U test results for independent variables

Independent variable	APC median	Non-APC median	Median difference	*p* value
IMD score	18.1	21.5	–3.4	< 0.001
List size	10,569	8408	2161	< 0.001
Females proportion	0.504	0.502	0.002	< 0.001
Age: Children (proportion)	0.101	0.102	–0.001	0.071
Age: Adolescents (proportion)	0.115	0.116	–0.001	0.017
Age: Adults (proportion)	0.585	0.584	–0.001	0.395
Age: Older adults (proportion)	0.191	0.181	0.01	0.007
Ethnicity: White (proportion)	0.982	0.968	0.014	< 0.001
Ethnicity: Mixed (proportion)	0.01	0.0	0.01	< 0.001
Ethnicity: Asian (proportion)	0.0	0.01	–0.01	< 0.001
Ethnicity: Black (proportion)	0.0	0.0	0.0	< 0.001
Ethnicity: Other (proportion)	0.0	0.0	0.0	< 0.001

APC practices had a statistically significantly lower IMD score (M_diff_ = -3.4, *p* < 0.001) than non-APC practices (i.e., APC practices were established in less socioeconomically deprived areas), and APC practices had a significantly larger list size (M_diff_ = 2161, *p* < 0.001). APC practices also had a slightly higher proportion of females (M_diff_ = 0.002, *p* < 0.001). A Chi-squared test showed that there was no significant difference in the urban-rural split of APC and non-APC practices (χ^2^ = 1.916, *p* = 0.1663).

In terms of age categories, the difference in the proportion of children and adults between the two groups was not statistically significant. APC practices had a significantly lower proportion of adolescents (M_diff_ = -0.001, *p* = 0.017), and a significantly higher proportion of older adults (M_diff_ = 0.01, *p* = 0.007), compared to non-APC practices.

In terms of ethnicity, APC practices had a higher proportion of White (M_diff_ = 0.014, *p* < 0.001) and Mixed (M_diff_ = 0.01, *p* < 0.001) patients, and a lower proportion of Asian (M_diff_ = -0.01, *p* < 0.001) patients, compared to non-APC practices.

The clearly visible structural differences in Fig. 2 between APC and non-APC practices highlight the necessity of controlling for sociodemographic confounders in our subsequent regression modelling.

#### Quality outcomes

**Fig. 3:**
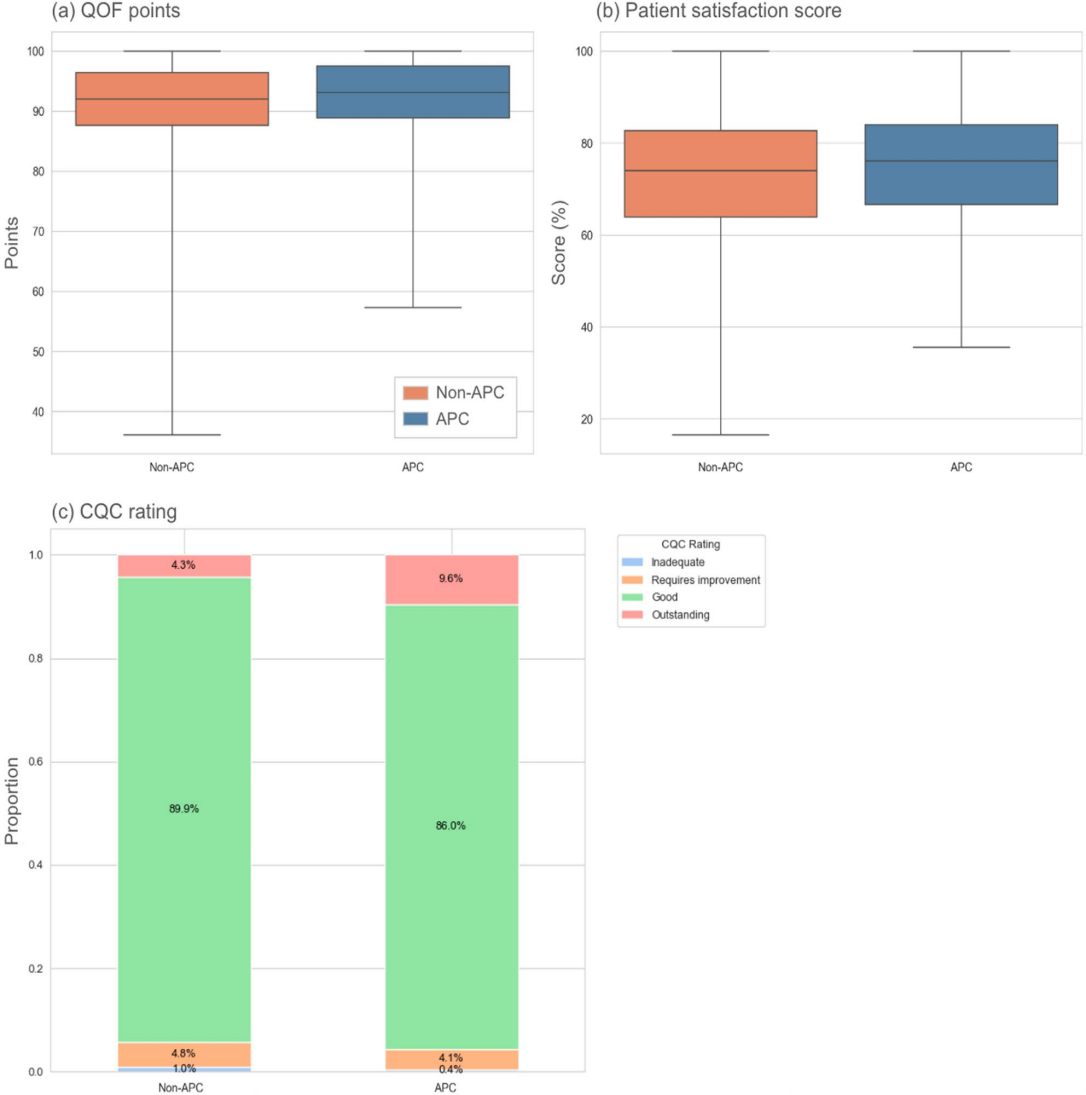
Distribution of outcome variables, comparing APC (blue) and non-APC (orange) practices: **a** QOF points; **b** patient satisfaction scores; **c** CQC rating. Box-and-whisker plots in panels (**a**) and (**b**) display median, upper and lower quartile as box, and minimum and maximum values as whiskers

Figure 3 displays the distribution of the three outcome variables used in this study. There were notable distributional differences between the groups: for example, the median patient satisfaction score for APC practices was visibly higher, with a tighter interquartile range suggesting more consistent performance. Since data in Fig. 2 indicate that APC practices also benefit from favorable structural characteristics (e.g. lower deprivation, larger list size), multivariable regression is required to determine if these performance gaps persist when controlling for these confounders.

Shapiro-Wilk tests on the outcome variables indicated significant non-normality (*p* < 0.001 in all three cases). However, our large sample size (*n* = 6,063) provides sufficient robustness against the violation of the normality assumption for regressions and T-tests, based on the Central Limit Theorem.

T-tests reveal that APC practices had a statistically significantly higher QOF score than non-APC practices (*t* = 3.445, *p* = 0.001), as well as a significantly higher patient satisfaction score (*t* = 3.613, *p* < 0.001). Chi-squared testing showed that APC practices also had a significantly higher CQC rating than non-APC practices (χ^2^ = 33.350, *p* < 0.001), with a notably larger proportion of APC practices achieving an “Outstanding” rating (Fig. 3c).

### Multivariable regression modelling

Table 3 summarises the main effects modelling results for the three outcome variables.

**Table 3:** Results from main effects multivariable regression modelling (without moderation)

	QOF points(OLS)	Patient satisfaction score(OLS)	CQC rating(ordinal logit)
Statistic		Model fit	
Observations, N	6063	6063	6063
Adjusted R^2^	0.068	0.163	–
AIC	42,250	47,880	5188
Variable	β (95% CI)		Odds Ratio (95% CI)
APC status	0.893 (*p* = 0.009) ** (0.219, 1.567)	1.979 (*p* < 0.001) *** (0.918, 3.040)	1.321 (*p* < 0.001) *** (1.159, 1.506)
IMD score	–0.105 (*p* < 0.001) *** (–0.125, − 0.086)	–0.154 (*p* < 0.001) *** (–0.184, − 0.123)	0.998 (*p* = 0.362) (0.994, 1.002)
List size	–0.001 (*p* < 0.001) *** (–0.001, − 0.001)	–0.001 (*p* < 0.001) *** (–0.001, − 0.001)	1.000 (*p* = 0.002) ** (1.000, 1.000)
Urban	–0.154 (*p* = 0.627) (–0.776, 0.468)	–2.976 (*p* < 0.001) *** (–3.956, − 1.997)	0.779 (*p* < 0.001) *** (0.689, 0.880)
Prop. female	25.438 (*p* < 0.001) *** (15.689, 16.266)	0.308 (*p* = 0.969) (–-15.045, 15.662)	0.178 (*p* = 0.063) (0.029, 1.096)
Prop. older adults	12.295 (*p* < 0.001) *** (8.325, 16.266)	21.459 (*p* < 0.001) *** (15.206, 27.713)	0.779 (*p* < 0.001) *** (0.689, 0.880)
Prop. White ethnicity	-0.705 (*p* = 0.182) (-1.740, 0.331)	7.782 (*p* < 0.001) *** (6.151, 9.412)	1.335 (*p* = 0.005) ** (1.091, 1.634)

Coefficients for OLS Models represent the change in the outcome variable for a one-unit increase in the predictor

Odds Ratios (OR) for the CQC Model represent the factor change in the odds of achieving a higher CQC rating

Significance: *** *p* < 0.001; ** *p* < 0.01; * *p* < 0.05

#### QOF points

The OLS model explained a small proportion of variation (adjusted R^2^ = 0.068). APC accreditation was associated with a 0.89 point increase in QOF (*p* = 0.009). Higher socioeconomic deprivation (IMD) was linked to lower QOF performance (0.11 QOF score decrease per IMD point, *p* < 0.001). All other variables were significant except geography and White ethnicity, with list size showing only a very small positive effect.

#### Patient satisfaction

The OLS model explained a relatively larger proportion of variation in patient satisfaction rate (adjusted R^2^ = 0.163). APC accreditation was significantly associated with a 1.93%-point increase in patient satisfaction (*p* < 0.001). Higher socioeconomic deprivation was associated with reduced patient satisfaction (0.15%-point decrease per IMD point, *p* *<* 0.001), while urban practices scored 2.98% point lower compared to rural practices (*p* < 0.001). Practices with a higher proportion of older adults and White ethnicity tended to have higher satisfaction rates (*p* < 0.001).

#### CQC rating

APC accreditation was associated with a 32.1% odds increase of achieving a superior CQC rating (*p* < 0.001). Urban locations reduced the odds by 22.1%, compared to rural locations (*p* < 0.001), practices with a higher proportion of older adults and White ethnicity were associated with a 94.7% and 33.5% higher odds, respectively, of achieving a superior CQC rating (*p* < 0.001).

To understand any potential effects of the temporal discrepancies between the APC data and some of the CQC ratings, we conducted an additional exploratory sensitivity analysis stratifying the sample by rating date. Since the APC was launched in 2018, we stratified the sample by CQC rating pre-2018 and post-2018. Among practices rated before the APC launch in 2018 (*n* = 2,755), future APC accreditation was not significantly associated with CQC ratings (OR = 1.220, *p* = 0.094). In contrast, for practices rated from 2018 onwards (*n* = 3,349), accreditation was significantly associated with higher CQC ratings (OR = 1.391, *p* < 0.001).

#### Summary

Across all models, APC accreditation was consistently associated with better performance, most notably for patient satisfaction, as were practices with higher proportions of older adults. By contrast, higher socioeconomic deprivation and urban location were generally associated with less favourable outcomes.

### Social equity analysis

The moderation analysis is presented in Table 4. For all three models, the added interaction term (APC x IMD score) was not statistically significant, suggesting that the relationship between APC status and QOF performance was uniform across the socioeconomic spectrum. While the addition of the interaction term resulted in APC status no longer being significant in the QOF and CQC models, the non-significance of the interaction term suggests we should default to interpreting the simpler (non-moderated) models as the basis for our primary findings.

**Table 4:** Results from multivariable regression modelling, with moderation

Variable	QOF points (OLS)	Patient satisfaction score (OLS)	CQC rating (ordinal logit)
	β (95% CI)		Odds Ratio (95% CI)
APC × IMD score	-0.026 (*p* = 0.416) (-0.088, 0.036)	–0.028 (*p* = 0.575) (-0.126, 0.070)	1.008 (*p* = 0.175) (0.996, 1.020)
APC status	1.433 (*p* = 0.055) (-0.031, 2.897)	2.564 (*p* = 0.029) * (0.259, 4.870)	1.113 (*p* = 0.453) (0.841, 1.473)
IMD score	-0.103 (*p* < 0.001) *** (-0.123, -0.083)	–0.1516 (*p* < 0.001) *** (–0.183, − 0.120)	0.998 (*p* = 0.221) (0.994, 1.001)
List size	–0.0001 (*p* = 0.06) ** (–0.001, − 0.001	–0.0004 (*p* < 0.001) *** (–0.001, − 0.001)	1.000 (*p* = 0.002) ** (1.000, 1.000)
Urban	-0.161 (*p* = 0.611) (-0.784, 0.461)	–2.984 (*p* < 0.001) *** (–3.964, − 2.004)	0.780 (*p* < 0.001) *** (0.690, 0.883)
Prop. female	25.448 (*p* < 0.001) *** (15.699, 35.197)	0.319 (*p* = 0.967) (–15.035, 15.674)	0.179 (*p* = 0.063) (0.029, 1.100)
Prop. older adults	12.252 (*p* < 0.001) *** (8.280, 16.224)	21.412 (*p* < 0.001) *** (15.156, 27.669)	1.979 (*p* = 0.087) (0.905, 4.329)
Prop. White ethnicity	-0.698 (*p* = 0.187) (-1.733, 0.338)	7.789 (*p* = 0.575) (6.159, 9.420)	1.332 (*p* = 0.005) ** (1.088, 1.630)

Significance: *** *p* < 0.001; ** *p* < 0.01; * *p* < 0.05

Results from the stratification analysis to test the independent effect of APC status on each outcome (controlling for other covariates) within each IMD decile (decile 1 = most deprived, decile 10 = least deprived) are summarised in Table 5. They show that the effect of APC status on QOF achievement was statistically significant only in deciles 3 and 8, where APC practices were associated with QOF scores that are 2.6 points and 2.2 points higher, respectively, compared to non-APC practices. For all other deciles, APC status had no reliable effect in the model for QOF points.

**Table 5:** Results from decile stratification analysis

Decile	QOF points (APC coefficient)	Patient satisfaction score (APC coefficient)	CQC rating (Odds ratio, 95% CI)
1 (most deprived)	-0.754 (*p* = 0.623)	-0.109 (*p* = 0.959)	1.017 (*p* = 0.941) (0.644, 1.607)
2	-0.777 (*p* = 0.616)	2.354 (*p* = 0.300)	1.811 (*p* = 0.024) * (1.083, 3.032)
3	2.606 (*p* = 0.046) *	2.263 (*p* = 0.230)	2.069 (*p* = 0.003) ** (1.289, 3.323)
4	-0.967 (*p* = 0.548)	0.621 (*p* = 0.783)	1.390 (*p* = 0.225) (0.817, 2.367)
5	0.791 (*p* = 0.458)	4.322 (*p* = 0.05) *	1.517 (*p* = 0.042) * (1.016, 2.264)
6	1.543 (*p* = 0.171)	-1.061 (*p* = 0.541)	1.263 (*p* = 0.261) (0.840, 1.898)
7	-0.641 (*p* = 0.550)	0.020 (*p* = 0.989)	1.050 (*p* = 0.715) (0.715, 1.542)
8	2.177 (*p* = 0.023) *	5.365 (*p* = 0.001) ***	1.705 (*p* = 0.003) ** (1.203, 2.417)
9	0.215 (*p* = 0.828)	-0.271 (*p* = 0.871)	0.969 (*p* = 0.889) (0.621, 1.513)
10 (least deprived)	-0.773 (*p* = 0.384)	1.700 (*p* = 0.236)	0.916 (*p* = 0.646) (0.629, 1.333)

Coefficients represent the change in the outcome variable for a one-unit increase in the predictor

Odds Ratios for the CQC Model represent the factor change in the odds of achieving a higher CQC rating

Significance: *** *p* < 0.001; ** *p* < 0.01; * *p* < 0.05.

For patient satisfaction, the effect was statistically significant only in deciles 5 and 8, where APC practices were associated with satisfaction scores 4.3 points and 5.4 points higher, respectively, compared to non-APC practices. There was no significant relationship between APC status and patient satisfaction in all other deciles.

The CQC results show that APC status was associated with statistically significantly increased odds of a higher CQC rating in the mid-range of socioeconomic deprivation (deciles 2, 3, 5 and 8). In particular, APC accreditation in decile 3 was associated with a doubling of the odds of achieving a higher CQC rating. It had no effect at the extreme ends of the socioeconomic deprivation scale.

##  Discussion

This is the first study to explore the relationships between health outcomes and a national PA programme for health run through English primary care. Practices accredited through the APC initiative were less likely to be located in a socioeconomically deprived area, had larger list sizes and had higher proportions of older adults in their patient demographics.

APC practices tended to have higher outcomes across all three of the selected quality metrics, compared with non-APC practices. Compared to CQC rating and patient satisfaction score, the impact of APC accreditation on QOF points was relatively marginal, with only a 0.89-point increase. It should be noted, however, that our sample of QOF scores ranged from 73 points to 100 points, so the small increase nonetheless represents about 3.3% of the sample range. A potential explanation for this small effect is that there are currently no standard QOF indicators to reward the promotion of PA. As a result, any association may be linked to mediating factors such as organisational efficiency.

Patient satisfaction and CQC are more subjective measures, which may reflect a practice’s desire to deliver more holistic care, inclusive of PA promotion. Our sensitivity analysis of the association of APC accreditation with CQC ratings pre- and post-2018 suggests that the strong positive association observed in our main model was driven principally by ratings awarded during the APC period. This weakens any hypothesis of reverse causality (i.e. that practices rated as ‘Outstanding’ in CQC are simply more likely to sign up to the APC), suggesting instead that the factors driving accreditation are concurrent with those driving recent regulatory success.

Previous studies show that higher CQC ratings are closely linked to greater funding, based on practice list size [27]. This may help explain why larger practices are more likely to hold APC accreditation, although many other factors may also play a role. Workforce issues are particularly important, as higher GP density is associated with better patient experience in England [28]. There is likely a complex interplay between workforce factors (including factors such as GP density, morale, culture and leadership ). Previous research has highlighted that APC accreditation may be linked with improved staff morale and culture [16], but further work is required to explore the impact of these organisational factors on practice outcomes and the directionality of impact.

It is also well recognised that practices located in more socioeconomically deprived areas often face higher patient demand and greater resource limitations – an effect known as the ‘inverse care law’ [22]. Despite this concept first being described in 1971 [29], systematic inequities in healthcare delivery persist [30], making it difficult to implement voluntary programmes equitably, including the APC [22]. These inequities may be exacerbated by patients from more socioeconomically deprived areas being less likely to be referred to – and take part in – PA programmes, as demonstrated for the Welsh National Exercise Referral Scheme [31], which operates at an individual patient level as opposed to a practice level.

Evidence from social prescribing, a comparable programme in the UK healthcare system, demonstrates that a targeted approach can improve the equity of reach. While we did not directly analyse social prescribing mechanisms in this study, they offer a lens through which to understand the implications of how targeted support can improve equity of reach in voluntary programs. A recent review of social prescribing services by Bu et al. [32] highlighted that, through targeted provision of resources, there was an equity of referral across different socioeconomic groups. Bu et al. [32] also found that individuals from more socioeconomically deprived areas, younger adults, men, and ethnic minorities were more likely to be referred to social prescribing via non-medical routes (e.g. charities, schools and religious centres). This highlights an opportunity to develop APC resources to be better integrated into non-medical contexts [33].

When compared with non-APC practices, we found that APC practices had statistically significantly lower IMD scores, higher proportions of older adults and a higher proportion of White ethnicity. This implies evidence of socioeconomic inequities in the take-up of the APC programme across English GP practices. However, our moderated regression models showed no evidence of inequity among practices that implemented the APC, since the model effects were consistent across socioeconomic groups. Our subsequent decile stratification analysis suggested that APC accreditation may be linked to higher outcomes in the mid-range deciles (i.e. not at the extremes), but given the lack of interaction at the global level, these localised effects must be interpreted with caution.

Our modelling also revealed the presence of urban-rural inequality. Despite prior research highlighting poorer care provision in rural areas [34, 35], we found that urban practices were strongly associated with a 2.98%-point penalty in patient satisfaction score, and a lower CQC rating (22.1% lower odds). This highlights the need to focus on optimising the delivery of the APC in urban geographies.

### Policy implications

We now turn to discussing the potential implications of our findings for UK healthcare policy. While our study was not designed to isolate causality and make definitive statements about the APC’s role in driving quality improvements, it provides evidence that the APC could play an important role in shaping policy discussions concerning the integration of PA promotion and social prescribing initiatives. With only 8.6% of practices in England signed up to the APC as of September 2025, there is some distance to go before “movement as medicine” [36] is embedded within GP practices across the country to support the 10-Year Health Plan for England [37].

Our results provide evidence for some level of inequity in the takeup of the APC, which should guide the development of targeted support, resources and tailored implementation strategies for practices in more socioeconomically deprived communities. This could include dedicated funding streams, enhanced training and bespoke support networks to help overcome resource and workload constraints. In an era of fiscally constrained healthcare provision in the UK [38], decisions around deploying voluntary accreditation programmes such as the APC should be guided by social equity concerns.

Future research should seek to identify any potential barriers practices face in fully implementing APC best practices, and to propose robust mechanisms to support practices in embedding preventative health measures, particularly in underserved communities. The issue of causality needs to be addressed by conducting randomised controlled or natural experiment studies on the deployment of specific components of the APC [39]. This would help to isolate the driver of observed quality improvements; for example, does the APC lead to improved outcomes because it targets patients, or because it targets staff? Or is there an entirely unrelated process at play? Qualitative research that engages directly with key initiative partners (e.g. practice staff, patients) could reveal how implementation of the APC varies by sociodemographic context, and the mechanistic ways in which different uptake strategies affect outcomes.

### Study limitations

We acknowledge several limitations to our study. First, we employed an observational study design, allowing us to identify statistical associations but not to establish causality. The possibility of confounding by unmeasured variables remains inherent. For instance, practices that voluntarily pursue APC status might inherently possess characteristics such as proactive leadership, better internal resources, and/or a pre-existing strong commitment to health promotion that are not captured by publicly available data. These unmeasured factors could predispose them to better quality outcomes regardless of APC accreditation, leading to residual confounding despite statistical controls for measured confounders.

Second, the voluntary nature of the APC initiative introduces a potential for selection bias. Practices that choose to engage with and achieve APC status may already be more motivated, innovative, or better-resourced than non-accredited practices. This self-selection could mean that observed positive outcomes are partly attributable to these underlying characteristics rather than solely to the APC initiatives themselves. While we applied statistical adjustments for practice characteristics in our models, we may well have missed factors that drive a practice’s decision to pursue accreditation.

Third, while the outcome measures we selected – QOF points, patient satisfaction rate, and CQC ratings – serve as valuable proxies for “quality outcomes”, they may not fully capture the entire spectrum of benefits associated with PA promotion initiatives within a primary care setting. For example, the long-term impacts on patient health behaviours or community engagement might not be fully reflected in these quantitative metrics.

Fourth, the temporal alignment of our datasets was not perfect, meaning that our datasets were somewhat spread in time. A handful of practices had not been rated by CQC since 2015, which predates the establishment of the APC.

Fifth, the IMD dataset is sometimes criticised in health research because it includes a health domain [40]. However, health is only a small component of the index, so we consider it suitable as an independent variable in our models.

Finally, we treated APC status as a simple binary measure (i.e. accredited vs. not accredited), and we did not assess the degree or quality of APC implementation within accredited practices. In reality, the implementation of any programme is likely affected by a variety of factors, including resource constraints, time/workload and communication gaps [41]. This lack of granularity in implementation fidelity may have obscured the true relationship between effective APC adoption and quality outcomes, and that the modelled effects are more strongly tied to the extent of implementation rather than merely the status of accreditation.

## Conclusions

Our findings reveal that APC accreditation was associated with improved outcomes across all three quality indicators, although other sociodemographics factors also had statistically significant effects. This is relevant given the growing emphasis on prevention across the political spectrum, and new national policies seeking to prioritise prevention. However, findings from this study suggest that practices opting into APC accreditation may already be better resourced, since they are larger and serve less socioeconomically deprived patient populations.

Moderation analysis highlighted uniform effects across the socioeconomic deprivation spectrum, and thus no evidence of inequity in the implementation of the APC. Followup stratification analysis revealed that the positive effects on patient satisfaction were concentrated in the mid-range of socioeconomic deprivation, but these localised effects should be interpreted with caution.

While causality and inequity could not be ascertained using our observational study method, there is a possibility that the voluntary APC could inadvertently widen existing quality gaps in patient experience. To ensure equitable access to “movement as medicine”, APC accreditation could be accompanied by targeted funding and tailored support for practices in highly socioeconomically deprived areas, enabling them to embed PA promotion while reducing persistent inequities.

## Figures and Tables

**Figure:**
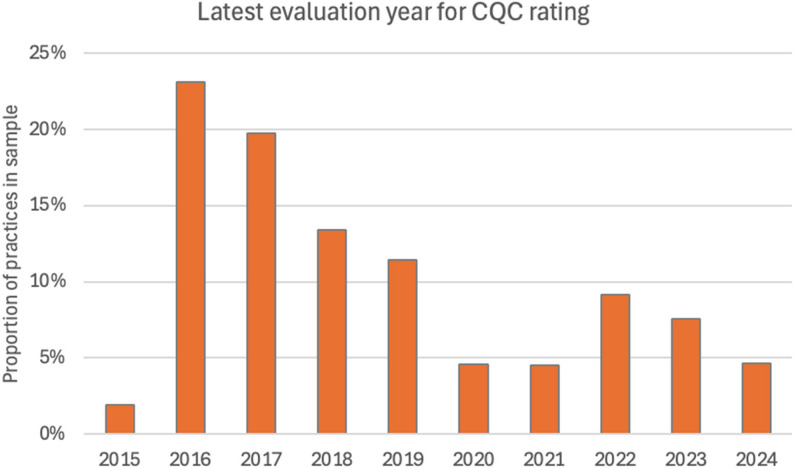
Distribution of CQC evaluation year

**Figure:**
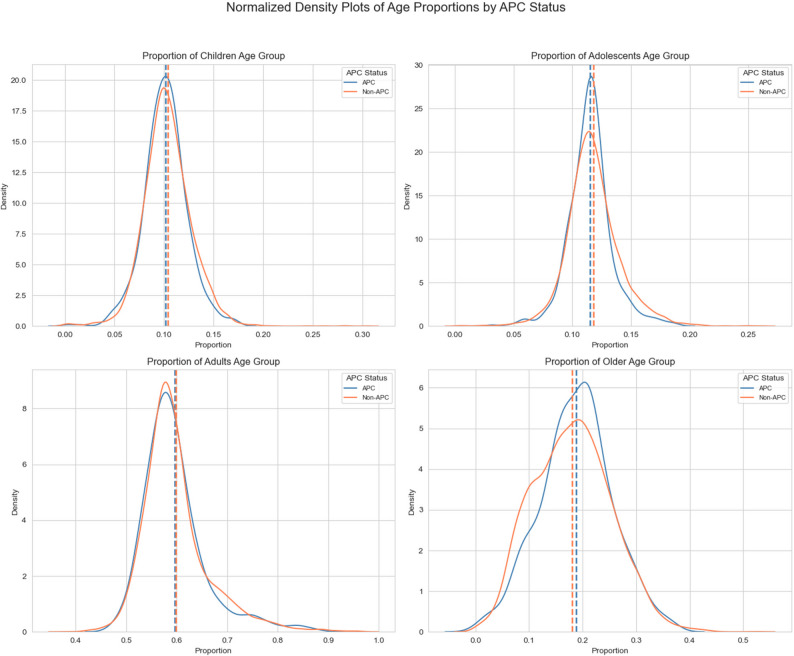
Distribution of age proportions by APC status

## Data Availability

All analysis code and core data files can be made available upon request. They can all be found on the following public access Github repository: (https://github.com/jeromemayaud/apc-general-practice-outcomes).

## Abbreviations

APC: Active Practice Charter
CQC: Care Quality Commission
GP: General practice
GPPS: General practice patient survey
IMD: Index of Multiple Deprivation
LSOA: Lower Layer Super Output Area
NHS: National Health Service
OHID: Office for Health Improvement and Disparities
OLS: Ordinary Least Squares
OR: Odds Ratios
PA: Physical Activity
QOF: Quality and Outcomes Framework
RCGP: Royal College of General Practitioners
RUC: Rural Urban Classification
VIF: Variance inflation factor
